# The emerging importance of the α-keto acid dehydrogenase complexes in serving as intracellular and intercellular signaling platforms for the regulation of metabolism

**DOI:** 10.1016/j.redox.2024.103155

**Published:** 2024-04-10

**Authors:** Ryan J. Mailloux

**Affiliations:** School of Human Nutrition, Faculty of Agricultural and Environmental Sciences, McGill University, Ste-Anne-de-Bellevue, Quebec, Canada

## Abstract

The α-keto acid dehydrogenase complex (KDHc) class of mitochondrial enzymes is composed of four members: pyruvate dehydrogenase (PDHc), α-ketoglutarate dehydrogenase (KGDHc), branched-chain keto acid dehydrogenase (BCKDHc), and 2-oxoadipate dehydrogenase (OADHc). These enzyme complexes occupy critical metabolic intersections that connect monosaccharide, amino acid, and fatty acid metabolism to Krebs cycle flux and oxidative phosphorylation (OxPhos). This feature also imbues KDHc enzymes with the heightened capacity to serve as platforms for propagation of intracellular and intercellular signaling. KDHc enzymes serve as a source and sink for mitochondrial hydrogen peroxide (mtH_2_O_2_), a vital second messenger used to trigger oxidative eustress pathways. Notably, deactivation of KDHc enzymes through reversible oxidation by mtH_2_O_2_ and other electrophiles modulates the availability of several Krebs cycle intermediates and related metabolites which serve as powerful intracellular and intercellular messengers. The KDHc enzymes also play important roles in the modulation of mitochondrial metabolism and epigenetic programming in the nucleus through the provision of various acyl-CoAs, which are used to acylate proteinaceous lysine residues. Intriguingly, nucleosomal control by acylation is also achieved through PDHc and KGDHc localization to the nuclear lumen. In this review, I discuss emerging concepts in the signaling roles fulfilled by the KDHc complexes. I highlight their vital function in serving as mitochondrial redox sensors and how this function can be used by cells to regulate the availability of critical metabolites required in cell signaling. Coupled with this, I describe in detail how defects in KDHc function can cause disease states through the disruption of cell redox homeodynamics and the deregulation of metabolic signaling. Finally, I propose that the intracellular and intercellular signaling functions of the KDHc enzymes are controlled through the reversible redox modification of the vicinal lipoic acid thiols in the E2 subunit of the complexes.

## 1 Overview of the KDHc enzymes

**Fig. 1:**
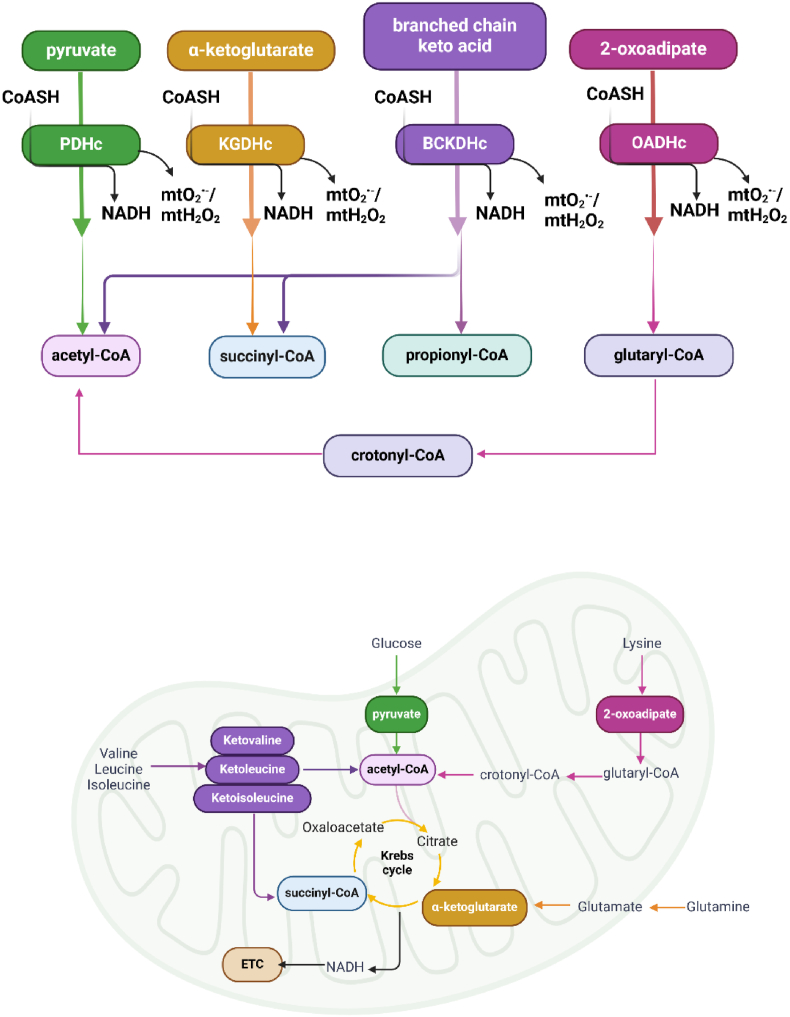
**Schematic depiction of the α-ketoacid dehydrogenase complexes (KDHc) metabolism.** Pyruvate dehydrogenase complex (PDHc; green), α-ketoglutarate dehydrogenase complex (KGDHc; orange), branched chain keto acid dehydrogenase complex (BCKDHc; purple), and 2-oxoadipate dehydrogenase complex (OADHc; mauve) use the α-keto acids, pyruvate, α-ketoglutarate, branched chain keto acids (BCKAs) and 2-oxoadipate as substrates to form acetyl-CoA, succinyl-CoA, propionyl-CoA, and glutaryl-CoA. The α-keto acids are formed from the metabolism of monosaccharides, glutamate, and glutamine (as well as other amino acids), branched chain amino acids (valine, leucine, and isoleucine), and lysine. Note that ketoleucine, ketoisoleucine, and ketovaline generate acetyl-CoA, propionyl-CoA, or succinyl-CoA. The acyl-CoAs generated by these reactions feed into the Krebs cycle to form intermediates for other anabolic reactions or are used to generate NADH, which injects electrons into the electron transport chain (ETC) to drive ATP biosynthesis. (For interpretation of the references to colour in this figure legend, the reader is referred to the Web version of this article.)

The α-ketoacid dehydrogenase complexes (KDHc) occupy critical metabolic checkpoints in bioenergetics by connecting monosaccharide, amino acid, and fatty acid metabolism to mitochondrial ATP production (Fig. 1) [1,2]. Although each KDHc fulfills a distinct catabolic role, the four dehydrogenases in this family of enzymes share common features in terms of their structure, overall composition, catalytic mechanism, regulation by allosteric factors, covalent modifications, and control by ions and the products they form. The oxidative decarboxylation of KDHc substrates, pyruvate (PDHc), α-ketoglutarate (KGDHc), branched-chain keto acids (BCKA; BCKDHc), and 2-oxoadipate (OADHc), respectively, results in the formation of acyl-CoAs and NADH. The acyl-CoAs are oxidized further by the Krebs cycle and NADH produced by the KDHc injects electrons into the electron transport chain (ETC) through complex I to drive OxPhos [[3], [4], [5], [6], [7], [8]]. PDHc connects glycolysis to the Krebs cycle by converting pyruvate formed by monosaccharide metabolism into acetyl-CoA (Fig. 1) [9]. The acetyl-CoA is then converted to citrate through a Claisen-type condensation reaction catalyzed by citrate synthase in the presence of oxaloacetate. The amino acids Ala, Gly, Cys, Ser, and Thr also form pyruvate through transamination reactions, making PDH an important point for the intake of certain amino acids into the Krebs cycle [10]. PDHc is heavily regulated by a kinase (PDHK) and phosphatase (PDP). Cells contain several PDK isoforms (PDK 1–4) which are expressed in specific tissues. PDK1-4 inhibits PDHc in response to hypoxia, nutrient deprivation, and calorie restriction, and when fatty acid (FAO) rates are high [11]. Notably, defects in PDK function has been implicated in the progression of several diseases like diabetic cardiomyopathy, cancer, thrombosis, and cholestasis [[12], [13], [14]]. Thus, there is high therapeutic potential in the targeting of PDK for the treatment of several diseases.

KGDHc is the fourth enzyme in the Krebs cycle, and it catalyzes the oxidative degradation of α-ketoglutarate to succinyl-CoA (Fig. 1). The succinyl-CoA is then metabolized further by the Krebs cycle, producing ATP through succinyl-CoA synthetase, which forms succinate, the substrate for succinate dehydrogenase (complex II) of the ETC [15,16]. KGDHc is vital for amino acid metabolism because it is required for glutamate biosynthesis, the main source and sink for amines in cells. Glutamate catabolism is mediated by glutamate dehydrogenase (GDH), which couples its reversible oxidative deamination to the formation of α-ketoglutarate [17,18]. Notably, glutamate is also used as an amide source for the biosynthesis of other amino acids. For example, glutamate is used to produce aspartate from oxaloacetate, a reaction catalyzed by aspartate aminotransferase (AAT). This forms α-ketoglutarate, which is metabolized by KGDHc. Thus, KGDHc activity is vital for amino acid homeostasis and the biosynthesis of cell proteins. Glutamate is also a neurotransmitter that modulates various neural functions and therefore KGDHc activity is integral for sustaining neuronal signaling. Defects in KGDHc can cause glutamate excitotoxicity, a type of cell death triggered by excessive glutamate production by neurons and glial cells [19]. Glutamate is also an important fuel for cancer cell hyper-proliferation and survival [20]. Cancer cells rewire glutamate/glutamine and Krebs cycle metabolism to promote tumorigenesis [21]. This metabolic reconfiguration is facilitated, in part, by the transcription factor c-myc, an important oncogenic driver that promotes glutamate/glutamine degradation [22].

BCKDHc is required for the degradation of branched chain amino acids (BCAA) leucine (Leu), isoleucine (Ile), and valine (Val) (Fig. 1) [23]. BCAA metabolism begins when branched chain amino acid transferase (BCAT) transaminates α-ketoglutarate to form the corresponding branched-chain keto acids (BCKA), ketoleucine, ketoisoleucine, and ketovaline [24]. These BCAAs are then oxidized by BCKDHc generating acetyl-, propionyl-, and/or succinyl-CoA, respectively [25]. Genetic defects in the E1β subunit of BCKDH causes a rare autosomal recessive disease called Maple Syrup Urine Disease (MSUD), which is characterized by deficiencies in BCAA degradation and the accumulation of BCKAs in the urine [25]**.** BCAA catabolism is also an interorgan pathway that involves communication between hepatocytes and other tissues like skeletal muscle [26]. The liver is a principal site for BCKA metabolism in mammals [26]. However, BCAAs must first be converted to BCKAs in other tissues like muscle due to the absence of BCAT in hepatocytes [27]. Recent studies have shown defects in liver BCKDHc activity in the liver shifts BCKA oxidation to the muscle and other organs, resulting in BCAA accumulation and the disruption of lipid metabolism [26]. This leads to glucose intolerance, heart failure, and obesity [28,29]. Importantly, BCAA accumulation due to defective BCKDHc results in the inhibition of pyruvate carrier in mitochondria and the suppression of gluconeogenesis in hepatocytes [26]. This likely contributes to the development of metabolic and cardiovascular diseases listed above. Like PDHc, BCKDHc is modulated by its phosphorylation and dephosphorylation, reactions that are mediated by branched chain ketoacid dehydrogenase kinase (BDK) and phosphatase PPM1k (aka PP2Cm) [30]. BDK deactivates BCKDHc whereas PPM1k has the opposite effect [31]. Notably, the accumulation of BCAAs is also a marker for tumorigenesis, which has been linked to the overstimulation of BDK [32]. Thus, there is high therapeutic potential in targeting the regulation of BCKDHc for the treatment of cancers, cardiovascular diseases, and metabolic diseases.

OADHc, also known as DHTKD1, is required for the mitochondrial degradation of lysine and converts 2-oxoadipate to glutaryl-CoA and NADH [33]. The glutaryl-CoA is metabolized to crotonyl-CoA by glutaryl-CoA dehydrogenase (GCDH), which is then converted to acetyl-CoA [34]. Genetic studies have linked mutation in the *Dhtkd1* gene, which encodes the E1 subunit of OADH, to the development of several neurological disorders and the onset of a disease called alpha-ketoadipic aciduria [35]. Impaired OADH is also linked to insulin resistance, cardiovascular disease risks, and Charcot-Marie-Tooth neuropathy [36]. Like other acyl-CoAs, glutaryl-CoA is used for the “*glutarylation*” of proteinaceous lysine residues, which can be reversed by the NAD^+^-dependent deglutarylase activity of sirtuins like Sirt5 in mitochondria [37]. Notably, reversible glutarylation has been linked to regulation of proteins in many tissues and has even been suggested to be neuroprotective [38,39]. Furthermore, defects in protein glutarylation, either due to inborn errors in OADH activity and/or loss of Sirt5, has been linked to insulin resistance, cardiovascular diseases, and neurological disorders [39]. Sirt5 is a well-known NAD^+^-dependent deacetylase. However, it also possesses desuccinylase and deglutarylase activity meaning it is required for the modulation of overall lysine acylation status, which is discussed in more detail below [40].

## 2 Structure and catalytic mechanism of KDHc

**Fig. 2:**
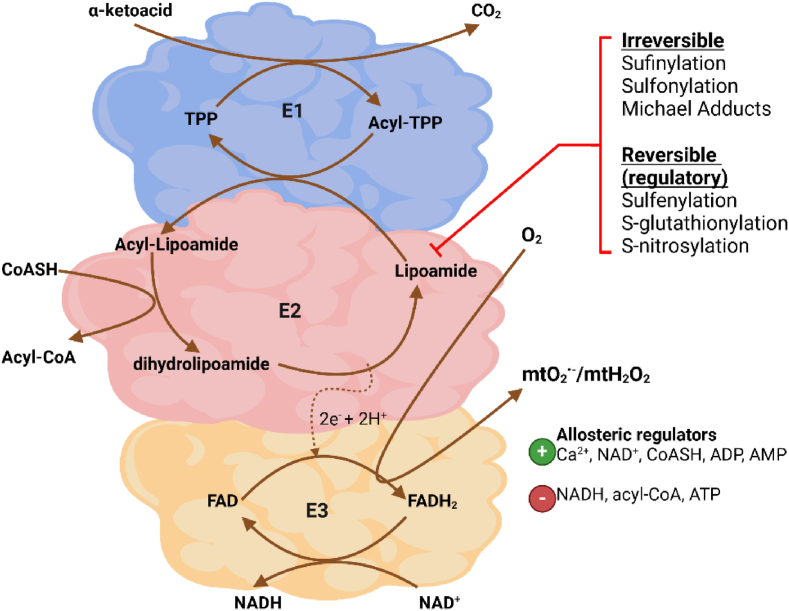
**The catalytic cycle for the α-ketoacid dehydrogenase complexes (KDHc).** The α-ketoacid is first decarboxylated on the C1 position by the α-ketoacid decarboxylase (E1) subunit, transferring the acyl group to thiamine pyrophosphate (TPP). The dihydrolipoamide acyltransferase (DLAT, E2) transfers the acyl-group from the TPP to the lipoic acid. The E2 subunit then catalyzes a thiol disulfide exchange reaction in the presence of coenzyme A (CoASH) forming a new thioester linkage with the acyl group on the lipoic acid, generating acyl-CoA. This results in dihydrolipoamide formation. The dihydrolipoamide is then oxidized by the dehydrogenase activity of the E3 subunit (dihydrolipoamide dehydrogenase: DLD or E3), reducing FAD to FADH_2_ and then producing NADH. Electrons leak from the FAD through side reactions that generates semiflavin radicals, flavin hydroperoxides, and oxy-flavin radicals, which form mitochondrial superoxide (mtO_2_^•-^) and/or mitochondrial hydrogen peorxide (mtH_2_O_2_). Formation of either mtO_2_^•-^ or mtH_2_O_2_ depends on the redox state of the flavin radical intermediates and its interactions with molecular oxygen (O_2_). The mO_2_^•-^ is dismutated to mtH_2_O_2_ by superoxide dismutase (matrix = SOD2 isozyme). The activity of KDHc and its mtO_2_^•-^/mtH_2_O_2_ can be reversibly inhibited and then reactivated by redox modifications like sulfenylation, S-glutathionylation, and S-nitrosylation of the vicinal thiols in the lipoic acid residue of the E2 subunit. The enzymes can be irreversibly deactivated through the formation of adducts like sulfinylation and sulfonylation or covalent modification with lipid peroxidation end products. The KDHc are also activated and deactivated, respectively, by allosteric regulators like Ca^2^, NAD^+^, CoASH, and ADP, respectively (green +) and NADH, acyl-CoA, NADH, or ADP (red -). (For interpretation of the references to colour in this figure legend, the reader is referred to the Web version of this article.)

The KDHc enzymes are composed of the same basic subunits: E1 (α-ketoacid decarboxylase; KDC), E2 (dihydrolipoyl acyltransferase; DLAT), and E3 (dihydrolipoamide dehydrogenase; DLDH) (Fig. 2). However, they are distinct from one another in terms of the number of copies and orientation of the three subunits that are used to form the multimer complexes. PDHc has a stoichiometry for the E1:E2:E3 subunits of 40:40:20, which forms a multisubunit holoenzyme that is ∼9.5 MDa in size [41]. By contrast, KGDH is predicted to contain ∼12 E1 and ∼12 E3 subunits surrounding a 24-mer E2 core, forming a multisubunit complex that is ∼3.2 MDa [40]. BCKDHc contains ∼6–12 copies of the E1 and E3 subunits organized around a 24-meric cubic E2 core [42]. OADHc is also comprised of multiple copies of the E1, E2, and E3 subunits but it is unclear how many of the three subunits are required to form the multi-subunit holoenzyme. Another distinguishing feature of the KDHc enzymes is only PDHc and BCKDHc are modulated by kinases and phosphatases. The kinases and phosphatases that are used by mammalian cells to deactivate and activate, respectively, PDHc and BCKDHc were briefly described above and were discussed in detail in several recent reviews [24,43,44]. KGDH harbors an adaptor protein called KGD4 that was reported to be required for the assembly of the subunits into a functional complex [45]. KGDHc is targeted for phosphorylation in *Corynebacterium glutamicum* by serine/threonine protein kinase G (PknG) [46]. However, there is currently no evidence showing mammalian KGDHc is controlled by reversible phosphorylation. Similarly, it is unknown if OADHc is regulated by phosphorylation.

The catalytic cycle for the KDHc enzymes is summarized in Fig. 2. A variety of co-factors and prosthetic groups are utilized by KDHc to catalyze the multistep conversion of the α-keto acid to acyl-CoA and NADH. These cofactors and prosthetic groups are thiamine pyrophosphate (TPP), lipoic acid, CoASH, and NAD^+^ [47]. The conversion of α-keto acid to acyl-CoA and NADH is initiated by the decarboxylase activity of the E1 subunit. Here, the α-keto acid is bound and used to covalently modify the TPP. This drives the oxidative decarboxylation of the α-keto acid, forming an acylated TPP intermediate (Fig. 2) [48]. The acyl group is then transferred from the E1 subunit to the oxidized lipoic acid on the E2 subunit, creating a thioester linkage between the acyl group and the vicinal thiols of the lipoate. The acyltransferase activity of the E2 subunit then facilitates a thiol disulfide exchange reaction between the acyl-lipoic acid and CoASH [1,47]. This reduces the vicinal sulphydryl groups in lipoic acid, releasing acyl-CoA. The reducing equivalents in the dihydrolipoamide are then transferred to the tightly bound FAD ^+^ group located in the E3 subunit. This forms FADH_2_, which is then used by the E3 subunit to reduce NAD^+^ to NADH (Fig. 2) [10]. Together, the catalytic cycle of the E1, E2, and E3 subunits of the KDHc enzymes involves several acyl transfer steps coupled to the cycling of sulphydryl groups and flavins between different oxidation states. Notably, these redox active functionalities imbue the KDHc enzymes with the unique characteristic to sense the surrounding redox environment through covalent modification or oxidation of its vicinal lipoic acid sulfurs or serve as a source of reactive oxygen species (ROS), specifically mitochondrial superoxide (mtO_2_^•-^) and mitochondrial hydrogen peroxide (mtH_2_O_2_), which is formed by the FAD center in the E3 subunit [1,49]. These chemical features make the KDHc vital for the mitochondrial redox sensing and signaling system (discussed further below).

**Fig. 3:**
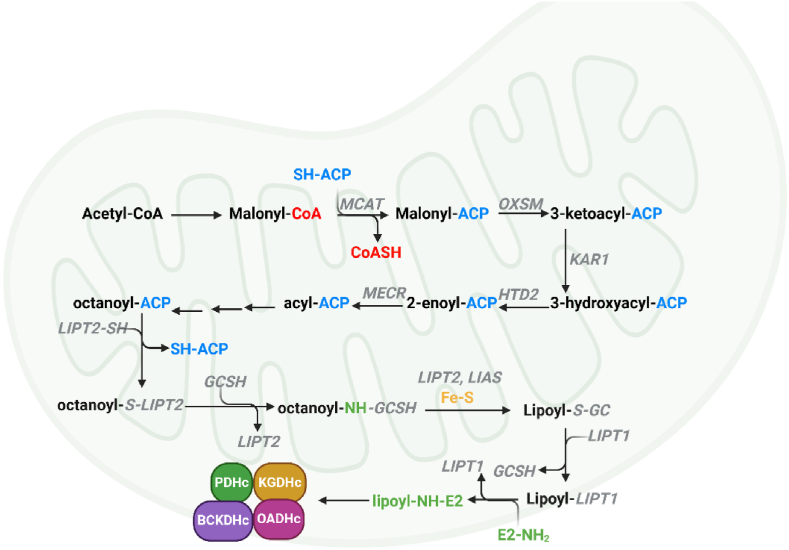
**A schematic representation of the mitochondrial fatty acid synthesis (mtFAS) pathway and the biosynthesis of lipoic acid for its covalent addition to the E2 subunit of α-ketoacid dehydrogenase complexes (KDHc).** The generation of malonoyl-CoA results in its transfer to acyl-carrier protein (SH-ACP) by maloyl-CoA:ACP transferase (MCAT). The malonoyl group is oxidized by 3-ketoacyl-ACP synthase (OXSM) and then reduced by ketoacyl-ACP reductase (KAR1) to produce 3-hydroxyacyl-ACP. This is then acted on by 3-hydroxyacyl-thioesterase (HTD2) and then enoyl-thioester reductase to form octanoyl-ACP. The octanoyl group is then transferred to the active site of octanoyl-transferase (LIPT2), which is then moved to Glycine H protein (GCSH). It is here the octanoyl group is thiolated in the presence of Fe–S and S-adenosylmethionine (SAM). Amidotransferase (LIPT1) transfers the lipoate to the Lys residue in the E2 subunit of the KDHc.

Mitochondria are well-known for facilitating nutrient breakdown to produce ATP by oxidative phosphorylation (OxPhos). However, these double membraned organelles of endosymbiotic origin are also important biosynthetic hubs that express many important evolutionarily conserved enzymes required for the genesis of macromolecules. This includes a conserved mitochondrial fatty acid synthesis (mtFAS) pathway that is integral for the genesis of lipoic acid and other longer fatty acids [50,51]. Why mitochondria need a lipogenesis pathway is still unclear since the organelle actively takes up many fatty acids. However, it is possible it is required because mitochondria are hubs for sulphur homeostasis and the mtFAS-mediated biosynthesis of lipoate requires sulphur from Fe–S clusters [52,53]. Furthermore, several studies that knocked out genes encoding the components of mtFAS identified mitochondrial lipogenesis is vital for bioenergetics and tissue function [54,55]. This has also sparked discussions surrounding the role of defective mtFAS in the manifestation of metabolic diseases like metabolic dysfunction-associated fatty liver disease (MAFLD), obesity, and type 2 diabetes mellitus (T2DM). The KDHc family of enzymes is highly unique for many reasons, one of which is their activity relies on the conjugation of lipoic acid to the E2 subunit of the enzyme complex. This lipoic acid is formed by the mtFAS pathway (Fig. 3). Lipoic acid (6,8-dithiooctanoic acid) was first identified in the 1950s and was later found to be synthesized by mtFAS [56,57]. The mtFAS pathway is like the bacterial type II FAS system (FASII), which is made up of individual enzymes that catalyze separate reactions in lipid biosynthesis (Fig. 3) [51,58,59]. This contrasts with the FASI system in the cytoplasm of mammalian cells where the individual reactions in lipogenesis are catalyzed by a multifunctional protein that carries out all the steps of fatty acid biosynthesis [[60], [61], [62]]. The pathway involves the diversion of acetyl-CoA formed by PDHc towards the genesis of malonyl-CoA (Fig. 3). This is followed by the transfer of the malonyl moiety to mitochondrial acyl carrier protein (mtACP) [[63], [64], [65]]. The resulting malonyl-mtACP is then elongated two carbons at a time through the sequential production of 3-keto-acyl-mtACP, 3-hydroxy-acyl-mtACP, and enoyl-mtACP, eventually generating octanoyl-mtACP (Fig. 3) [59,64,65]. Octanoyl-mtACP is then *trans*-sulphurated by Fe–S dependent lipoyl synthase (LIAS) in the presence of S-adenosyl methionine (SAM) to produce lipoyl-mtACP [57,59,65,66]. This is then transferred to the newly synthesized KDHc. mtFAS in general is vital for the function of mitochondria since the lipids derived from this pathway are key for the function of mitoribosomes, RNA processing, respiratory complex assembly, and Fe–S biosynthesis [59,64,65,67]. Importantly, defects in PDHc function may contribute to mitochondrial dysfunction by limiting the availability of acetyl-CoA building blocks required to maintaining mtFAS activity. This could manifest into the progression of many disease states like cardiovascular, neurological and metabolic disorders, which are related to defects in mitochondrial metabolism and function. Further, it would be prudent to ascertain if other acetyl-CoA sources like amino acid catabolism or ketone body breakdown can contribute to mtFAS if PDHc activity is nullified. Collectively, mtFAS is a highly important, yet overlooked, pathway that is key in sustaining the activity of KDHc and many other mitochondrial functions.

The products of the reactions catalyzed by the KDHc family of enzymes serve as potent allosteric regulators for these complexes (Fig. 2). The state of the [NADH]/[NAD^+^] and [acyl-CoA]/[CoASH], which is dictated by KDHc activity, Krebs cycle flux, and the turnover of NADH and/or acyl-CoA, can serve as a powerful regulator of KDHc activity. A high [NADH]/[NAD^+^] or [acyl-CoA]/[CoASH] ratio inhibits the activities of KDHc enzymes whereas a decrease in the ratio has the opposite effect [1,[68], [69], [70]]. Maintenance of a low [NADH]/[NAD^+^] and [acyl-CoA]/[CoASH] ratio is dependent on complex I activity (or the activity of an NADH-metabolizing alternative oxidase) and the activities of enzymes that break the thioester linkage in acyl-CoAs (e.g. citrate synthase or succinyl-CoA synthetase), respectively [1,[68], [69], [70]]. Other allosteric regulators include the [ATP]/[ADP] and [AMP], which control the activity of the KDHc enzymes in response to the energy needs of the cell. Calcium (Ca^2+^) also activates PDHc and KGDHc when in the 0.1–10 μM range [71]. Importantly, these same allosteric factors also control the activities of the kinases and phosphatases that inhibit or induce the KDHc enzymes. A high [ATP]/[ADP], [NADH]/[NAD^+^], or [acyl-CoA]/[CoASH] ratio activates PDHc and BCKDHc kinase [72]. This results in PDHc or BCKDHc phosphorylation, inhibiting the enzyme complex.

## 3 KDHc are mtO_2_^•-^/mtH_2_O_2_ sources and redox sensors that produce intracellular and intercellular signaling molecules

### 3.1 KDHc enzymes generate mtO_2_^•-^/mtH_2_O_2_

The capacity of the KDHc enzymes to generate mtO_2_^•-^/mtH_2_O_2_ has been discussed in several excellent reviews [1,10,47,49,73]. It was first reported by Massey et al., in 1969 that dihydrolipoamide dehydrogenase (E3 subunit) can produce mtO_2_^•-^ during the reverse transfer of electrons from NADH [74,75]. Later renewed interest in the reactions led to the discovery KGDHc can produce a mixture of mtO_2_^•-^/mtH_2_O_2_ in synaptosomes [19,73,[76], [77], [78], [79]]. Notably, it was found KGDHc formed considerable quantities of mtO_2_^•-^/mtH_2_O_2_ during forward electron transfer (FET) from α-ketoglutarate, but also in a reverse reaction with NADH (reverse electron transfer; RET) [19,73,[76], [77], [78], [79], [80]]. This intrinsic ability of PDHc and KGDHc to generate mtO_2_^•-^/mtH_2_O_2_ through electron side reactions was later confirmed using either recombinant complexes of bacterial or porcine heart origin or in muscle, liver, and heart tissue mitochondria [48,[81], [82], [83], [84]]. At this point it was conventionally thought complex I and III of the ETC were the primary sources of mtO_2_^•-^/mtH_2_O_2_. However, these seminal studies established that mtO_2_^•-^/mtH_2_O_2_ can be generated by mitochondrial dehydrogenases external to the ETC. These first studies also demonstrated that under certain experimental conditions, KGDHc, and potentially other KDHc enzymes, are potent sources of mtO_2_^•-^/mtH_2_O_2_ and can display rates of generation that are higher than complex I and equal to complex III [19,[73], [74], [75], [76], [77], [78], [79], [80]]. The principle site for mtO_2_^•-^/mtH_2_O_2_ generation in the KDHc is thought to be the FAD group in E3 subunit, but genesis has also been reported to occur through the formation of thiamine pyrophosphoryl radical (TPP•) and thiyl radical (-S•) in the E1 and E2 subunits, respectively [1]. It is important to point out, however, experiments with recombinant PDHc and KGDHc or liver mitochondria from C57BL6N mice have shown blocking the vicinal thiols of the lipoic acid residue in the E2 subunit by S-glutathionylation or S-nitrosylation nullifies mtO_2_^•-^/mtH_2_O_2_, supporting the notion that the FAD center in DLD is the main source in KDHc [84,85]. Experiments conducted with the purified E3 subunit and purified PDHc and KGDHc of porcine heart origin confirm the main source of mtO_2_^•-^/mtH_2_O_2_ is the FAD center [74,75,[81], [82], [83],^86^]. Studies that used permeabilized rat skeletal muscle mitochondria, intact and permeabilized mouse liver mitochondria, cultured macrophages, and heart mitochondria has revealed PDHc, KGDHc, BCKDHc, and OADHc display rates of mtO_2_^•-^/mtH_2_O_2_ greater than complex I [1,49,83,84,[86], [87], [88], [89]]. Horvath et al. reported KGDHc is also an important mtO_2_^•-^/mtH_2_O_2_ generator in mouse brain tissue when the ETC is operating under RET conditions [90]. In this case, mitochondrial fuels like succinate and glycerol-3-phosphate, which by-pass the Krebs cycle and donate electrons directly to coenzyme Q_10_ (CoQ) in the ETC, generate mtO_2_^•-^/mtH_2_O_2_ by KGDHc by producing NADH at complex I [90]. Together, a significant amount of evidence has been generated demonstrating the KDHc family of enzymes are main mtO_2_^•-^/mtH_2_O_2_ sources in mammalian mitochondria.

Competitive inhibitors for PDHc and KGDHc, 3-keto-2-methylvaleric acid (KMV) or CPI-613 (devimistat), have been used to show both enzyme complexes are potent mtO_2_^•-^/mtH_2_O_2_ generators [87,91]. Succinyl phosphonate, a selective inhibitor for KGDHc, also nullifies mtO_2_^•-^/mtH_2_O_2_ production by this enzyme complex [87]. KMV is a structural analog of α-ketoglutarate and potent inhibitor of KGDHc. CPI-613 is a lipoic acid analog used to inhibit the E2 subunit of all KDHc enzymes. Both inhibitors have been employed to successfully show KGDHc is a high-capacity site for mtO_2_^•-^/mtH_2_O_2_ generation. Recently, KMV was also used to show KGDHc is an important site for mtO_2_^•-^/mtH_2_O_2_ generation during the oxidation of fatty acids in liver mitochondria [92]. Notably, use of the new complex I and complex III inhibitors, S1QEL 1.1 (S1) and S3QEL 2 (S3), demonstrated that both ETC enzymes are low capacity sites for mtO_2_^•-^/mtH_2_O_2_ generation [92]. By contrast, adding KMV to reaction mixtures with S1 and/or S3 almost abolishes mtO_2_^•-^/mtH_2_O_2_ production during fatty acid oxidation [92]. It is important to emphasize here that the ETC is thought to be the main, if not only, source of mtO_2_^•-^/mtH_2_O_2_ during fatty acid oxidation. However, the new results published by Grayson et al. argue it is KGDHC, not the ETC, that is the chief mtO_2_^•-^/mtH_2_O_2_ supplier in liver mitochondria during fatty acid oxidation or when other carbon sources like pyruvate are used to fuel the Krebs cycle [92]. Together, this study provides new insight into the mechanisms of lipotoxicity and how it triggers disease progression through the induction of oxidative distress. It must be acknowledged that the experiments discussed above were conducted exclusively with isolated mitochondria, synaptosomes and purified enzymes and do not quantify the contribution of KGDHc towards mtO_2_^•-^/mtH_2_O_2_ in cultured cells. Brand et al. has established the S1 and S3 compounds are important new tools for investigating mtO_2_^•-^/mtH_2_O_2_ generation in isolated mitochondria and cultured cells as both compounds selectively inhibit ROS generation by complexes I and III without interfering with respiration [93]. S1QEL was found to inhibit mtO_2_^•-^/mtH_2_O_2_ generation by complex I by 40–80 %, respectively, in isolated muscle mitochondria [93]. The S1 and S3 compounds were also successfully tested on cultured cells and used to show complexes I and III account for ∼50–90 % of the total mtO_2_^•-^/mtH_2_O_2_ production in C2C12, INS-1E, and HepG2 cells, respectively [[94], [95], [96]]. The S1QELs were also shown to prevent metabolic dysfunction associated fatty liver disease (MAFLD), glucose intolerance and insulin resistance in mice fed a high-fat diet by limiting complex I-mediated mtO_2_^•-^/mtH_2_O_2_ production [97]. However, it should be noted that Grayson et al. used S1 and S3 concentrations like the ones in these studies and found complexes I and III made only minor contributions to the overall mtO_2_^•-^/mtH_2_O_2_ producing capacity of the liver mitochondria. It was when KGDHc was inhibited that mtO_2_^•-^/mtH_2_O_2_ production was almost completely abolished. Further, Brand and colleagues did not assess the contribution of KGDHc towards the ROS production affected by the S1 and S3 compounds. It needs to also acknowledged that KMV is a substrate for BCKDHc since it is a product of isoleucine deamination. This KMV could be used by BCKDHc mtO_2_^•-^/mtH_2_O_2_ production. However, in several studies the KMV was shown to induce an ∼90 % decrease in mtO_2_^•-^/mtH_2_O_2_ generation in liver and muscle mitochondria oxidizing pyruvate, α-ketoglutarate, or glutamate with malate [87,92]. Together, this shows that studies investigating mtO_2_^•-^/mtH_2_O_2_ production should consider KGDHc and the other KDHc members as sources since these enzyme can be important ROS generators.

The KDHc family of enzymes are points of convergence for various fuels entering the Krebs cycle. As key metabolic checkpoints, the rate of mtO_2_^•-^/mtH_2_O_2_ generation by the KDHc enzymes can increase and decrease, respectively, in response to the efficiency of metabolite fluxes and electron transfer reactions in the Krebs cycle and the ETC. Limiting NAD^+^ *in situ* causes mtO_2_^•-^/mtH_2_O_2_ formed by PDHc and KGDHc to accumulate [78,86,87]. In addition, an increased [NADH]/[NAD^+^] or [acyl-CoA]/[CoASH] ratio also favors an increased rate of mtO_2_^•-^/mtH_2_O_2_ production in synaptic and permeabilized skeletal muscle mitochondria [78,86,87,98]. The observation that NADH accumulation can augment mtO_2_^•-^/mtH_2_O_2_ formation by KGDHc was demonstrated by Starkov et al. using rotenone, a complex I inhibitor [76]. Similarly, Horvath et al. successfully showed KGDHc can produce large quantities of mtO_2_^•-^/mtH_2_O_2_ when NADH is formed by the reversal of complex I, which can be prevented with rotenone [90]. Similar findings were made by Quinlan et al. where addition of rotenone significantly augmented mtO_2_^•-^/mtH_2_O_2_ production in rat muscle mitochondria oxidizing pyruvate or BCKAs [87]. Antimycin A and rotenone also drive up mtO_2_^•-^/mtH_2_O_2_ production by OADHc, which can be inhibited by ATP [88]. Lowering the levels of acetyl-CoA by activating carnitine acetyl-CoA transferase (CrAT), which decreases the [acetyl-CoA]/[CoASH] by forming acetyl-carnitine, limits mtO_2_^•-^/mtH_2_O_2_ production by PDHc [87,98]. Similarly, preventing the build-up of succinyl-CoA or depletion of α-ketoglutarate by adding aspartate to reaction mixtures decreases the rate of mtO_2_^•-^/mtH_2_O_2_ production by KGDHc [87].

The production of mtO_2_^•-^/mtH_2_O_2_ by the KDHc enzymes can be beneficial or have negative consequences for cells. In the context of benefits, the biosynthesis of mtO_2_^•-^/mtH_2_O_2_ is known to trigger cell oxidative eustress pathways, which are induced by the site-specific and reversible oxidation of protein cysteine switches (reviewed in Refs. [[99], [100], [101]]). This event can activate and deactivate key proteins required for the transmission of intracellular and intercellular redox signals to modulate gene expression, enzyme activities, ion transport, cell receptor function(s), or cell behaviors (e.g. motility or phagocytosis) [[99], [100], [101]]. A concentration of [mtH_2_O_2_] at ≤100 nM activates the eustress pathways that are vital for the regulation of many cell functions [[99], [100], [101]]. By comparison, [mtH_2_O_2_] at ≥ 100 nM has the opposite effect, inducing oxidative distress which is characterized by deregulated and nonspecific/irreversible protein cysteine oxidations and the onset of lipid peroxidation, cell death, and various pathologies [[99], [100], [101]]. The role of KDHc enzymes in activating beneficial eustress signals is not well defined. However, since enzymes like KGDHc are prominent mtO_2_^•-^/mtH_2_O_2_ generators, it would stand to reason they play a role in oxidative eustress. For instance, Long et al. recently showed mtH_2_O_2_ released by PDHc is integral for modulating the inflammatory response of cultured macrophages [89]. Other articles have postulated the KDHc enzymes contribute to oxidative eustress signaling because they display high rates of mtO_2_^•-^/mtH_2_O_2_ production [49,102]. The KDHc enzymes have also been implicated in promoting oxidative distress through the hyper-generation of mtO_2_^•-^/mtH_2_O_2_. As discussed above, the rate of mtO_2_^•-^/mtH_2_O_2_ production by the KDHc enzymes increases considerably when the [NADH]/[NAD^+^] and/or the [acyl-CoA]/[CoASH] ratios are increased. This suggests defects in NADH turnover by complex I, electron flow through the ETC, and/or disrupted Krebs cycle flux can amplify mtO_2_^•-^/mtH_2_O_2_ generation by KDHc enzymes causing oxidative distress, cell death, and disease progression. For instance, findings collected in the early 2000s led to the posit that defects in complex I can cause neurological diseases through the NADH-mediated induction of increased mtO_2_^•-^/mtH_2_O_2_ production by KGDHc [19,47,73,[76], [77], [78], [79]]. Interestingly, diminishing KGDHc is also protective against neuronal glutamate excitotoxicity, potentially through the prevention of mtO_2_^•-^/mtH_2_O_2_ generation [103,104]. By contrast, the overabundance of KGDHc has been implicated in the onset of obesity. Feeding rats an “obesogenic diet” and the onset of diet-induced obesity (DIO), which correlates with the induction of oxidative distress, is related to the increased expression of KGDHc [105]. The increased KGDHc in this case may be a response that is required to handle the increased acetyl-CoA caused by the free fatty acid overload of cells. However, this increased KGDHc may also contribute to the oxidative distress by promoting mtO_2_^•-^/mtH_2_O_2_ generation. By contrast, other studies have shown a deficiency in KGDHc can be detrimental to health and cause the progression of several diseases. KGDHc is partially depleted in brain tissue from neurological disorders [106,107]. This chronic decrease in KGDHc activity disrupts glucose utilization for ATP production by neurons, which correlates with a decline in mental function and the development of neurodegenerative disorders [[108], [109], [110]]. Deficiencies in the PDHc, BCKDHc, and OADHc have been linked to the development of neurological disorders and metabolic diseases like MAFLD, obesity, and diabetes [26,36,[111], [112], [113]]. Malnutrition also compromises the activity of KDHc, specifically due to the decreased availability of thiamine pyrophosphate and other cofactors critical for their activity [114]. For example, inadequate dietary intake of pantothenic acid or thiamine compromises KGDHc activity and succinyl-CoA biosynthesis, which can have adverse outcomes during pregnancy [114]. What has remained unclear is whether the decline in KGDHc can be harmful or protective to cells. Interestingly, Chen et al. proposed in one study that the short and temporary disabling of KGDHc enhances cell mtO_2_^•-^/mtH_2_O_2_ handling and mitigates the onset of oxidative distress whereas the long-term disruption of the enzyme complex has the opposite effect [104]. In this case, short-term inhibition was achieved with site-specific inhibitor for KGDHc, carboxyethyl succinyl phosphonate (CESP) [87,104,115]. Importantly, membrane penetrating trimethyl ester acetylphosphonate (AcPMe_2_), triethyl succinyl phosphonate (TESP) and trimethyl or triethyl adipoyl phosphonate (TEAP or TMAP) that selectively interfere with PDHc, KGDHc, and OADHc, respectively, were recently developed to evaluate the role of KDHc in triggering oxidative eustress and distress in cells [36,116,117]. These tools, like KMV and CPI-613, can induce a robust ∼80–90 % decrease in mtO_2_^•-^/mtH_2_O_2_ generation, making these inhibitors highly valuable in the study of KDHc enzymes in cell redox homeodynamics. Targeted disruption of PDHc and KGDHc with CPI-613 is now being explored as a means of treating several cancers as both enzyme complexes exhibit aberrant changes in activity during tumorigenesis [118]. Notably, it was recently suggested that MAFLD could be prevented through the temporary inhibition of PDHc and KGDHc using reversible redox modifications (reviewed in Ref. [102]). It is tempting to speculate the site-specific AcPMe_2_ and TESP inhibitors could prevent MAFLD through temporary PDHc and KGDHc inhibition. Also, mitochondria-targeted agents that induce temporary redox modifications, like mitochondria-targeted S-nitrosylation compound (MitoSNO), may be useful in protecting tissues from oxidative distress triggered by the KDHc enzymes.

### 3.2 KDHc enzymes are redox sensors

The use of mtO_2_^•-^/mtH_2_O_2_ in cell signaling requires that cells possess efficient mechanisms to prevent oxidative distress. This is fulfilled by antioxidant defenses that clear mtO_2_^•-^ and mtH_2_O_2_ to prevent the biosynthesis of highly reactive hydroxyl radicals that damage cell macromolecules [119,120]. Preventing mtO_2_^•-^/mtH_2_O_2_ buildup is also achieved through the redox sensing capabilities of ROS-generating enzymes [121]. In this context, ROS producers are deactivated following the oxidation of antioxidant pools like glutathione, after a burst in mtO_2_^•-^/mtH_2_O_2_ formation. This occurs through the reversible oxidation of specific cysteine thiols. For example, complex I is deactivated through the oxidation of key subunits, specifically NDUFS1 (Cys^531^ and Cys^704^) and ND3 (Cys^39^) [[122], [123], [124]]. The modification protects the vulnerable thiols from irreversible oxidation but also turns down mtO_2_^•-^/mtH_2_O_2_ generation to nullify oxidative distress and abrogate the progression of diseases [122,125,126]. In effect, the deactivation of mtO_2_^•-^/mtH_2_O_2_ production by mitochondrial generators of ROS serves as a self-contained negative feedback loop that is required to monitor cell redox status for the prevention of oxidative distress.

**Fig. 4:**
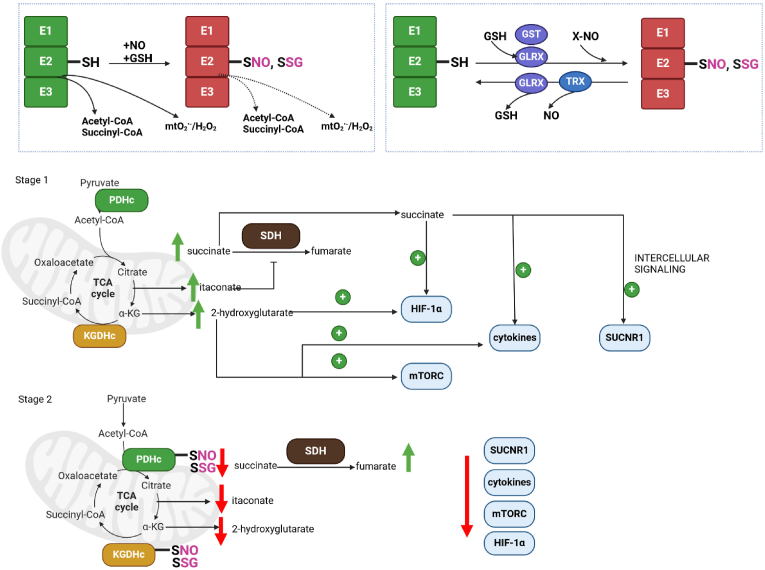
**Reprogramming of the Krebs cycle through reversible redox modification of PDHc and KGDHc is required to modulate immunometabolite availability for signaling.** Proposed role for reversible redox modifications S-glutathionylation and S-nitrosylation in the regulation of metabolic signaling. The top left panel enclosed in dashed lines depicts the impact of the reversible S-glutathionylation and S-nitrosylation of the vicinal lipoic acid thiols in the E2 subunit on mtO_2_^•-^/mtH_2_O_2_ production by KDHc enzymes. Note the S-glutathionylation and S-nitrosylation also inhibit KDHc activity. The top right panel enclosed by dashed lines depicts the reversible S-glutathionylation and S-nitrosylation of KDHc by glutaredoxins (Glrx), glutathione S-transferases (GST), transnitrosylation agents (X–NO; S-nitroso-glutathione, S-nitroso-CoA, nitro-proteins), and thioredoxins (TRX). The proposed mechanism is based on the studies that have identified S-glutationylation and S-nitrosylation as a key modulator for PDHc and KGDHc activity and mtO_2_^•-^/mtH_2_O_2_ and how the latter one controls the availability of important immunomodulatory metabolites (see text for a breakdown). The two-phase mechanism for controlling the availability of immunomodulatory metabolisms proposed here is based on the original proposal by Refs. [132,168]. In phase 1, the Krebs cycle is geared towards the biosynthesis of itaconate, which is formed from aconitate and the activity of aconitate decarboxylase, succinate, and 2-hydroxyglutarate. The itaconate is a powerful inhibitor of succinate dehydrogenase (SDH, complex II), resulting in succinate accumulation. This succinate activates intracellular and intercellular signals, respectively, through promotion of HIF-1α stabilization and cell-to-cell signaling through succinate receptor-1 (SUCNR1) activation. 2-hydroxyglutarate also accumulates activating HIF-1α and mammalian target for rapamycin complex 1 (mTORC1). For phase 2, the increased mtO_2_^•-^/mtH_2_O_2_ generation by PDHc and KGDHc (and other dehydrogenases) oxidizes cell glutathione pools and promotes nitric oxide production, resulting in the S-glutathionylation or S-nitrosylation of PDH and KGDH. This inhibits PDHc and KGDHc slowing Krebs cycle flux, limiting the formation of itaconate, succinate, and 2-hydroxyglutarate. It is likely BCKDHc and OADHc are regulated in a similar manner, which could also have signaling effects.

The lipoic acid residue in the E2 subunit of KDHc enzymes plays a key role in mitochondrial redox sensing and signaling. This redox sensing function of the KDHc, which has been identified for PDHc, KGDHc, and to a lesser extent BCKDHc, is depicted in Fig. 2 and in Fig. 4. The role of KDHc enzymes in redox sensing in mitochondria has also been discussed in several reviews [1,47,79]. The redox sensing activity of the KDHc is attributed to the reactivity of the vicinal thiols in the prosthetic group. KDHc-mediated redox sensing was first documented in 1978 when Sies and Moss discovered *tert*-butyl hydroperoxide interfered with oxidation of pyruvate and α-ketoglutarate, which could be prevented with glutathione peroxidase-4 [127]. It was later discovered PDHc and KGDHc are deactivated by lipid peroxidation end-product, 4-hydroxy-2-nonenal (4-HNE), and mtH_2_O_2_ [16,79,128,129]. This deactivation occurred through the redox modification of the vicinal thiols in the lipoic acid residue to the E2 subunit of PDHc and KGDHc [47,[130], [131], [132]]. Oxidation of the vicinal lipoic acid thiols in KDHc by mtH_2_O_2_ to a corresponding sulfenic acid (P–SOH) can also result in irreversible oxidation to corresponding sulfinic (P–SO_2_H) and sulfonic acids (P–SO_3_H) when mtH_2_O_2_ is high (Fig. 2) [129,133,134]. Modification by 4-HNE also forms an irreversible covalent adduct with the lipoic acid, which has been shown to be the underlying cause of defective Krebs cycle function and mitochondrial bioenergetics in the development of neurological disorders (Fig. 2) [[135], [136], [137]]. Inhibition of PDHc and KGDHc by irreversible oxidation is prevented by the reversible protein S-glutathionylation of the vicinal lipoic acid thiols (Fig. 4) [47,79,129,133,134]. Protein S-glutathionylation is a ubiquitous and reversible redox sensitive covalent modification that occurs through the addition and removal of a reduced glutathione (GSH) moiety to and from a proteinaceous cysteine thiol [[138], [139], [140], [141]]. The modifications are site-specific and enzymatically mediated by glutaredoxins (Glrx) and glutathione S-transferases and are required to modulate many cell functions, ranging from actin polymerization and immune cell function to muscle and heart contraction, neural activity, eye function, and liver metabolism [102,[142], [143], [144], [145], [146]]. In the context of KDHc regulation, the reversible glutathionylation prevents the further reaction of the P–SOH formed on the lipoic acid residue with mtH_2_O_2_ or 4-HNE, protecting the enzyme complexes. The deglutathionylase activity of Glrx2, the mitochondrial matrix glutaredoxin isoform, reverses the modification, reactivating the NADH-forming capacity of PDHc and KGDHc (Fig. 4) [133,147]. Most of the information collected to date on the modulation of KDHc enzymes by reversible redox modifications has been done by studying PDHc and KGDHc (and this has been reviewed extensively [47,102]). The exception is BCKDHc, which was recently found to undergo S-nitrosylation on its E2 lipoic acid residue [148]. However, in a separate study, it was shown BCKDHc does not undergo S-glutathionylation [149]. PDHc, KGDHc, and BCKDHc have also been found to undergo S-nitrosylation in several studies [131,132,150]. Like S-glutathionylation, the S-nitrosylation shuts down the activity of the KDHc but also likely protects the enzymes from irreversible deactivation through the overoxidation of the lipoic acid vicinal thiols. Further, thioredoxins (TRX), small oxidoreductases that catalyze disulfide exchange reactions, catalyze the denitrosylation of target proteins (Fig. 4) [151]. Defects in the redox regulation and redox sensing activities of PDHc and KGDHc has been suggested to play a role in the development of MAFLD and its advancement to metabolic dysfunction associated steatohepatitis (MASH) [102]. For example, prolonged deactivation of PDHc or KGDHc by S-glutathionylation due to oxidative distress has been linked to the development of DIO and MAFLD in mice fed a high-fat diet (HFD) [152]. The same study found promoting the deglutathionylation of mitochondrial proteins protects from DIO and MAFLD, likely through the activation of PDHc and KGDHc [152]. Finally, GLP-1 agonist liraglutide prevents the development of diabetic cardiomyopathy by promoting PDHc deglutathionylation, which enhances mycardial glucose clearance and oxidation [14].

### 3.3 Reversible redox modification of KDHc enzymes and the modulation of the availability of signaling metabolites

mtH_2_O_2_ has been shown to modulate several cell signaling pathways required for proliferation, differentiation, clonal expansion (of immune cells), adaptive signaling by triggering antioxidant and/or stress transcription factors (e.g. NRF2, HIF-1α, heat shock factor-1 (HSF-1), and NF-κB), maintenance of ion gradients, intracellular pH buffering, metabolic flux, anabolic reactions, and energy metabolism [[99], [100], [101],[153], [154], [155], [156]]. It is for this reason mtH_2_O_2_ is now considered a key second messenger in cells. In the context of intracellular signaling, mtH_2_O_2_ exerts its effects through the induction of site-specific and reversible oxidation of specific cysteines in proteins. For example, when cell oxidants are low, the antioxidant transcription factor NRF2 is targeted for proteasomal degradation by the oxidant sensor KEAP1 and E3 ubiquitin ligase Cullin-3 [157,158]. An increase in intracellular mtH_2_O_2_ triggers the oxidation of specific cysteines in KEAP1, targeting it for degradation, which causes NRF2 stabilization and migration into the nucleus where it stimulates the expression of antioxidant defense genes [159].

The reversible oxidation of cysteine switches in mitochondria controls mtO_2_^•-^/mtH_2_O_2_ generation [160,161]. mtO_2_^•-^/mtH_2_O_2_ generation by complex I was first found to be suppressed by reversible glutathionylation [122,162], which was later suggested to play an integral role in the propagation of redox signals through the cell [163]. The S-nitrosylation of complex I was later found to serve as a dampening device for mtO_2_^•-^/mtH_2_O_2_ generation during RET from succinate in the ETC [126,164]. This nitrosylation is cardioprotective since it negates oxidative distress through the suppression of mtO_2_^•-^/mtH_2_O_2_ production in response to ischemia-reperfusion injury [126,164]. These same factors make KDHc vital redox sensors as S-glutathionylation and S-nitrosylation also shut down mtO_2_^•-^/mtH_2_O_2_ biosynthesis by these enzymes. This also means the reversible S-glutathionylation and/or S-nitrosylation of the KDHc enzymes is crucial for the regulation of mitochondrial redox signaling. Indeed, oxidation of GSH pools because of high mtO_2_^•-^/mtH_2_O_2_ production in liver and cardiac mitochondria results in the S-glutathionylation of PDHc and KGDHc (reviewed in Ref. [102]). This suppresses mtO_2_^•-^/mtH_2_O_2_ genesis by both PDHc and KGDHc (reviewed in Ref. [102]). Protein S-nitrosylation was also recently found to be key for nullifying mtO_2_^•-^/mtH_2_O_2_ by PDHc and KGDHc as well [85,89]. Notably, Long et al. provided clear evidence the reversible S-glutathionylation of PDHc is vital for regulating macrophage activation in response to lipopolysaccharide (LPS) signaling [89]. PDHc is a pivotal metabolic target for LPS signaling and Long et al. showed this signaling involves reversible S-glutathionylation, which controls mtO_2_^•-^/mtH_2_O_2_ generation (Fig. 4) [89]. This results in the increased expression of pro-inflammatory cytokines, like IL-1β, and a decrease in anti-inflammatory ones, like IL-10 (Fig. 4) [89].

It has recently emerged that S-nitrosylation switches may also be required to modulate cell communication pathways by controlling the availability of critical signaling metabolites made by the Krebs cycle (Fig. 4). Indeed, PDHc, KGDHc, and BCKDHc are also targeted for reversible S-nitrosylation during the activation of macrophages [131,132]. In this context, the S-nitrosylation is vital for regulating the availability of different immunomodulatory metabolites, which has far reaching implications for the regulation of the innate immune system [131,132]. In this context, the reversible S-nitrosylation of PDHc, KGDHc, and BCKDHc controls the availability of immunomodulatory metabolites like succinate, itaconate, and 2-hydroxyglutarate [131,132]. The immunomodulatory function of succinate is mediated through the stabilization of HIF-1α and via intercellular signaling with G-protein coupled receptor-91 (GPCR91; succinate receptor (SUCNR1)) (Fig. 4) [165,166]. Availability of the succinate is controlled through the itaconate-mediated inhibition of succinate dehydrogenase [167]. The overlapping control over itaconate and succinate levels for signaling was suggested to be mediated through a “two-stage” metabolic reprogramming of the Krebs cycle (Fig. 4) [168]. In the first stage, itaconate and succinate accumulate to activate intracellular and intercellular signals. Stage 2 involves the S-nitrosylation of PDHc and KGDHc which shuts off both enzymes causing itaconate and succinate to dissipate [168]. This “two-stage” regulation has been linked to the control of macrophage activation through modulation of interferon-γ signaling [168]. 2-hydroxyglutarate availability is also controlled in this manner [131,132]. 2-hydroxyglutarate induces cell fate decision making in immune cells, such as naïve T cell differentiation, through regulation of the mTORC1 pathway, redox buffering capacity, and the induction of chromatin remodeling (reviewed in Ref. [169]) (Fig. 4). Of course, 2-hydroxyglutarate is also a powerful oncometabolite and thus perturbations in KGDHc S-nitrosylation, and perhaps S-glutathionylation, may also play a role in tumorigenesis [170]. It is important to emphasize here that mtO_2_^•-^/mtH_2_O_2_ is also required for immune cell regulation. Indeed, the availability of mtO_2_^•-^/mtH_2_O_2_ has been found to propagate oxidative eustress signals in macrophages and T cells for the induction of immune responses towards pathogens [171]. Thus, the reversible redox modulation of the KDHc enzymes may be an important platform for propagating intracellular and intercellular signals through control over mtO_2_^•-^/mtH_2_O_2_ and intracellular and intercellular signaling metabolites like succinate, itaconate, and 2-hydroxyglutarate (Fig. 4).

## 4 KDHc enzymes in the acyl modification of proteins

### 4.1 Acetylation

Acetylation is an important post-translational modification that integrates metabolic fluxes with the control of vital cell processes such as cell cycle progression, energy metabolism, gene expression, and circadian rhythms [172]. The link between metabolism and cell regulation is provided by acetyl-CoA and NAD^+^ which are required to drive the acetylation and deacetylation, respectively, of protein lysine residues [173]. The reactions are catalyzed by lysine acetylases (KATs) and deacetylases (KDACs), the latter of which is comprised of the NAD^+^-dependent Sirtuin (Sirt) family of proteins [174]. Histone lysine residues were uncovered to be the main targets for acetylation/deacetylation, which is vital for regulating nucleosomal dynamics and epigenomic programming [175]. In this case, genomic DNA is tightly packed in the nucleosome which consists of DNA nucleotide sequences wrapped around a histone octomer [175]. Histone acetylation increases the accessibility of the gene in the nucleosome, allowing for its transcription [176]. NAD^+^-independent and dependent deacetylation has the opposite effect.

**Fig. 5:**
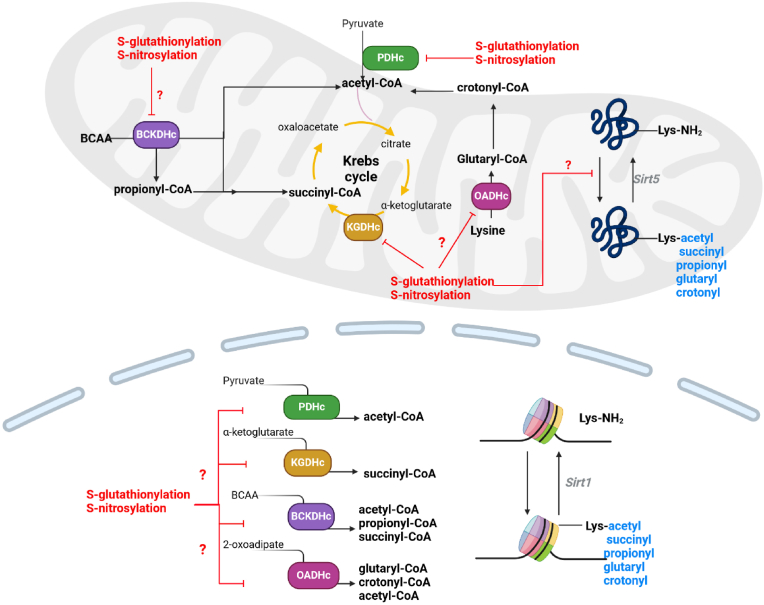
**The cellular acylation code is driven by KDHc activities in the mitochondria and nucleus**. PDHc, KGDHc, BCKDHc, and OADHc form acetyl-CoA, succinyl-CoA, propionyl-CoA, crotonyl-CoA, and glutaryl-CoA, which are used as substrates for the covalent modification of lysines (Lys) in the matrix of mitochondria or nuclear lumen. In mitochondria, the acylation of lysines is required to control bioenergetics. By contrast, the modifications play a role in the modulation of nucleosomal dynamics. The acylation of histone lysines or mitochondrial proteins is reversed by sirtuins (Sirt). Note there are 7 Sirt isoforms. Not all are listed in figure. Also, it is proposed that the redox reactions S-glutathionylation and S-nitrosylation are required to control the availability of the acyl-CoAs for Lys modifications. Thus, acylations are likely modulated by cell redox homeodynamics.

Provision of acetyl-CoA units for nuclear acetylation reactions has been shown to be mediated through a mitochondria-to-nucleus citrate shuttle mechanism [177]. Thus, metabolic support for the regulation of histone dynamics is supplied by PDHc, and possibly BCKDHc and OADHc (Fig. 1). Mitochondria are the main sites for acetyl-CoA generation in mammalian cells. PDHc and other pathways like fatty acid oxidation, ketone body degradation, and branched chain amino acid metabolism irreversibly generate acetyl-CoA, which then condenses with oxaloacetate in the presence citrate synthase to form citrate. The citrate then travels from mitochondria into the cytoplasm and/or nucleus through dicarboxylate antiporter solute carrier 25A1 (SLC25A1) where it is cleaved by ATP-citrate lyase (ACLY) to reform acetyl-CoA [178]. Notably, Sutendra et al. demonstrated the provision of acetyl-CoA units for histone acetylation is also mediated by a nuclear PDHc that binds to Histone-3 (H3) [179]. The PDHc occurs in the nucleus because of stimulation of cells by epidermal growth factor, which results in the acetylation of histones and induction of the cell cycle [179]. Most striking is the PDHc seems to “tunnel” from mitochondria to the nucleus after growth factor stimulation [179]. The channeling of PDHc to the nucleus was recently proposed to occur through the mitofusin-2 (MFN2)-mediated tethering of mitochondria to the nuclear envelope [180]. The PDHc crosses into the nucleoplasm by first forming a ring structure with lamin, which then allows it to penetrate through the nuclear envelope in a process that is independent of the nuclear pore complex [180]. It is still unclear exactly how PDHc, which is ∼9.5 MDa, crosses the nuclear envelope. However, the finding does provide a new and intriguing twist on nuclear metabolism. Other mitochondrial dehydrogenases like IDH-2 and MDH-2 were also recently found to have a cardioprotective effect after their translocation to the nucleus [181]. It is tempting to speculate BCKDHc and OADHc, which also form acetyl-CoA, migrate to the nucleus to elicit a similar effect on acetylation. It would also be of interest to interrogate the impact of redox modifications like S-glutathionylation and S-nitrosylation on nuclear acetyl-CoA production by PDHc. Indeed, it has been shown PDHK also migrates into the nucleus where it regulates acetyl-CoA genesis through PDHc phosphorylation [182]. Additionally, redox regulation may serve as a rapid means to modulate acyl-CoA availability for lysine modification. For example, S-glutathionylation and S-nitrosylation of nuclear PDHc in immune cells would negate acetyl-CoA production for nucleosomal regulation (Fig. 5). This could be reversed by glutaredoxin-2 splice variant b or c (Glrx2b or Glrxc), which localizes to the nucleoplasm [183]. Note that the Glrx2a variant localizes only to the matrix of mitochondria [183]. Likewise, the S-nitrosylation of PDHc in the nucleus can be reversed by nucleoredoxins. KDAT enzymes are also subjected to sulfenylation, which modulates nucleosome dynamics [184]. Although speculative, it is also likely the nuclear PDHc produces mtO_2_^•-^/mtH_2_O_2_ to facilitate the induction of nuclear redox signals that regulate transcription, chromatin structure and dynamics, and other pathways. It is also important to emphasize acetyl-CoA generated by PDHc in mitochondria also controls mitochondrial protein functions by lysine acetylation (Fig. 5). These lysine acetylations are reversed by NAD^+^-dependent Sirt5. In consideration of the fact that PDHc is a redox sensor, it is likely acetylation of mitochondrial proteins is modulated by the reversible S-glutathionylation and S-nitrosylation of PDHc (Fig. 5). Therefore, redox regulation of PDHc in the nucleus may be required to control nucleosomal dynamics whereas its reversible S-glutathionylation and S-nitrosylation in mitochondria regulates bioenergetics. This is achieved through the fine tuning of acetyl-CoA availability, which overlaps with mtO_2_^•-^/mtH_2_O_2_ production for signaling.

### 4.2 Succinylation, glutarylation, and propionylation in cell regulation and disease

Protein lysine residues can undergo many acylations including succinylation, propionylation, myristoylation, HMGcylation, glutarylation, malonylation, acetoacetylation, crotonylation, butyrylation, isobutyrylation, and α-methylbutyrylation [185]. Many of the acyl-CoA groups generated to drive these reactions are supplied by KGDHc (succinyl-CoA), BCKDHc (succinyl-CoA, propionyl-CoA, butyryl-CoA, isobutyryl-CoA, and α-methylbutyryl-CoA), and OADHc (crotonyl-CoA, glutaryl-CoA) (Fig. 1). The regulation of proteins through these protein lysine modifications in the nucleus and throughout the cell is still relatively understudied but recent reports have shown they are likely just as important as acetylation in the control of cell functions. Succinylation, for example, has emerged as a key posttranslational modification that is required to control various cell proteins. It has been shown the accumulation of succinyl-CoA, glutaryl-CoA, and the other acyl-CoAs listed above correlates with the development of several diseases since accumulation of these moieties can results in the spontaneous and non-specific lysine acylation. For example, the augmentation of the lysine glutarylation of mitochondrial proteins caused by a deficiency in GCDH, which is required to oxidize the glutaryl-CoA formed by OADHc, or increased OADHc activity is linked to the progression of neural and hepatic diseases and development of diabetes [36,186]. Notably, the site-specific OADHc inhibitor, TMAP, could mitigate some of these pathogological effects related to non-specific glutarylation [36]. Also, the same group predicted OADHc localizes to the nucleus, much like PDHc [187]. It would be interesting to examine the effect of TEAP or TMAP on the nuclear glutarylome and ascertain if glutaryl-CoA also serves as a critical coding device for nucleosome modulation. In fact, recent work has identified that KGDHc also localizes the nucleus and interacts with KAT2A to generate a histone H3 succinyltransferase complex [188]. Like glutarylation, succinylation was originally identified as an aberrant modification that occurs during the development of several diseases [3]. In this case, dysfunctional Krebs cycle metabolism causing succinyl-CoA build up leads to the spontaneous succinylation of mitochondrial proteins, exacerbating the metabolic dysfunction associated with stroke, neural disorders, and metabolic diseases [3]. However, in the case of nuclear KGDH-KAT2A, it is required to modulate gene expression, showing succinylation has a regulatory function for histones. The notion that succinylation regulates gene expression is reinforced by the histone desuccinylase activity of HDAC 1, 2 and 3, which are critical for promoter desuccinylation [189]. It is vital to also acknowledge that succinylation and glutarylation are required to modulate mitochondrial bioenergetics. For example, most succinylation targets are found in mitochondria where it regulates the urea cycle, ketogenesis, and Krebs cycle flux [190,191]. Notably, the conjugation of succinate to proteins is mediated by succinyl-CoA ligase (SCL), which is reversed in mitochondria by Sirt5. SCL deficiency due to recessive mutations in the gene encoding the enzymes causes mitochondrial encephalopathy, which is related to cell wide hyper-succinylation [192]. Lysine glutarylation is also required to control mitochondrial metabolism [191].

Like nuclear PDHc, it is unknown if nuclear KGDHc and OADHc are also modulated by S-glutathionylation and S-nitrosylation (Fig. 5). However, since both KGDHc and OADHc have also been reported to be important redox sensors, it is likely both nuclear isoforms are controlled in this manner (Fig. 5). Here, reversible redox modifications could serve as a means of regulating succinyl-CoA and glutaryl-CoA levels, impacting histone succinylation and glutarylation. In this context, the S-glutathionylation or S-nitrosylation of KGDHc or OADHc would decrease succinyl-CoA and glutaryl-CoA availability, impacting lysine acylation and potentially nucleosomal dynamics (Fig. 5). Conversely, deglutathionylation or denitrosylation of KGDHc or OADHc would have the opposite effect. It would be anticipated that if KGDHc or OADHc (and PDHc) are targeted for reversible redox modifications in the nucleus, that nucleoredoxins (e.g. Glrx2b/Glrx2c or Trx) would play a role in catalyzing their (de)glutathionylation and (de)nitrosylation. It is also worthy to point out KGDHc is also a major source of mtO_2_^•-^/mtH_2_O_2_. It is tempting to speculate that nuclear KGDHc and the other KDHc family members also produce nuclear O_2_^•-^/H_2_O_2_ for the modulation of redox signaling pathways in the nucleus. This would also mean the nuclear redox signaling functions of KGDHc and the other KDHc family members are also controlled by S-glutathionylation and S-nitrosylation and that the nuclear O_2_^•-^/H_2_O_2_ signals emanating from KGDHc and OADHc overlap with their lysine acylation functions.

Important also to the discussion on KDHc enzymes in modulation protein functions through lysine acylation is the implication of BCKDHc in supplying succinyl-CoA, propionyl-CoA, or acetyl-CoA for these reactions. Propionyl-CoA generated by BCKDHc activity has been proposed to be exported into the cytoplasm from mitochondria through its conversion to propionyl-carnitine [193]. The propionyl-carnitine is then converted back to propionyl-CoA in the cytoplasm for use in histone lysine propionylation in the nucleus [193]. The import of cytoplasmic propionyl-CoA into the nuclear space has been proposed to be facilitated by nucleoporin [193]. Thus, BCKDHc in mitochondria could modulate histone dynamics using propionyl-CoA. In a recent study, Yang et al. showed that BCAA metabolism-mediated production of propionyl-CoA led to the propionylation of histone 3 [194]. Surprisingly, Yang et al. found limiting BCAA metabolism lowered histone 3 propionylation, protecting the myocardium from damage [194]. This was related to increased expression of ETC components, lowered fibrosis and prevention of cardiac hypertrophy [194]. The authors speculated that the propionylation was due to the presence of BCKDH in the nucleus, but no evidence was supplied in this regard. To this point, a recent study showed cells compartmentalize propionyl-CoA metabolism, which is separated into cytoplasmic and nuclear propionyl-CoA pools [195]. In this case, only propionyl-CoA generated in the nucleus was suggested to result in lysine acetylation [195]. Thus, like PDHc and KGDHc, it is likely there is a nuclear BCKDHc isoform that is required for the regulation of nucleosomal dynamics. As described above, BCKDHc is also targeted for S-nitrosylation, which means the availability of propionyl-CoA in the nucleus may be modulated by reversible redox modifications. Finally, the acylations mediated by KGDHc, OADHc, and BCKDHc also occur in the mitochondrial matrix and are reversed by Sirt 5 (Fig. 5). Thus, it is highly probable the redox sensing activity of these KDHc also plays an integral role in dictating the degree of mitochondrial protein acylation through the modulation of acyl-CoA availability.

## 5 Conclusions and perspectives

The KDHc enzymes occupy pivotal metabolic intersections for the connection of monosaccharide, amino acid, fatty acid, and ketone body metabolism with catabolic and anabolic reactions vital for cellular health. The enzyme complexes are major hubs for metabolic regulation and are subjected to subtle control by allosteric modulators and covalent modifications that dictate metabolic fluxes in response to changing cell and physiological demands. The pathological significance of defects in KDHc function has been documented in many studies since chronic deactivation of these enzymes hampers mitochondrial bioenergetics and the provision of cellular ATP. However, defects in the activities of these enzymes and/or their regulation can have other far reaching consequences as KDHc serve as redox sensors that supply critical metabolites, ROS, and acyl-CoA molecules used in intracellular and intercellular signaling. In this review, I provide a new perspective on the critical signaling roles fulfilled by the KDHc enzymes by integrating their known functions as redox sensors with their newly identified duty in supplying metabolites and post-translational modifiers for the regulation of cell signaling pathways. There are still major deficiencies in understanding many of the redox sensing roles of the KDHc enzymes. Most work has focused on PDHc and KGDHc, but now BCKDHc and OADHc are emerging as critical redox sensors and mtO_2_^•-^/mtH_2_O_2_ producers. Further to this, there is still little information on the critical signaling functions fulfilled by the metabolites formed and degraded by the KDHc enzymes. However, as shown in this review, it is clear KDHc enzymes are vital signaling platforms that connect mitochondrial bioenergetics and redox sensing with cell regulation. Overall, more attention should be given to these enzymes since the KDHc are significant regulators of mitochondrial and nuclear metabolism and are required for the cell-wide regulation of metabolism.

## CRediT authorship contribution statement

**Ryan J. Mailloux:** Writing – review & editing, Writing – original draft, Funding acquisition, Conceptualization.

## Declaration of competing interest

The authors declare that they have no known competing financial interests or personal relationships that could have appeared to influence the work reported in this paper.

## Data Availability

No data was used for the research described in the article.
